# Clinical characterisation of exacerbations of severe eosinophilic asthma occurring on mepolizumab and placebo

**DOI:** 10.1183/23120541.00196-2024

**Published:** 2024-07-29

**Authors:** Imran Howell, Daniel J. Bratton, Gareth Hynes, Steven W. Yancey, Liam G. Heaney, Ian D. Pavord, Rahul Shrimanker

**Affiliations:** 1 Oxford Respiratory NIHR BRC and Respiratory Medicine Unit, Nuffield Department of Medicine, University of Oxford, Oxford, UK; 2 Clinical Statistics, GSK, Brentford, UK; 3 Respiratory Therapeutic Area, GSK, Research Triangle Park, NC, USA; 4 Department of Respiratory Medicine, Centre for Experimental Medicine, School of Medicine, Dentistry and Biomedical Sciences, Queen's University Belfast, Belfast, UK; 5 Bristol Academic Respiratory Unit, Bristol, UK

## Abstract

Mepolizumab, a monoclonal antibody targeting interleukin (IL)-5, depletes circulating eosinophils and reduces exacerbations of severe eosinophilic asthma [1]. However, some people continue to exacerbate potentially due to different, less corticosteroid-responsive mechanisms [2]. To characterise the changes in symptom and lung function seen in on-biologic exacerbations, we conducted a *post hoc* comparison of diary card data from prednisolone-treated exacerbations occurring during treatment with mepolizumab or placebo in three placebo-controlled trials [1, 3, 4].

## Shareable abstract

**Asthma exacerbations in people treated with mepolizumab result in less peak expiratory flow reduction than placebo but similar symptom scores. Symptoms recover slower, indicating these exacerbations may be less prednisolone responsive.** https://bit.ly/3xQsFRB

*To the Editor:*

Mepolizumab, a monoclonal antibody targeting interleukin (IL)-5, depletes circulating eosinophils and reduces exacerbations of severe eosinophilic asthma [1]. However, some people continue to exacerbate potentially due to different, less corticosteroid-responsive mechanisms [2]. To characterise the changes in symptom and lung function seen in on-biologic exacerbations, we conducted a *post hoc* comparison of diary card data from prednisolone-treated exacerbations occurring during treatment with mepolizumab or placebo in three placebo-controlled trials [1, 3, 4].

Diary card data were reviewed from three studies involving 1743 patients with severe eosinophilic asthma: DREAM [1], a 52-week study of three doses of mepolizumab (75, 250 or 750 mg intravenously, 4 weekly) *versus* placebo; MENSA [3], a 32-week study of two doses of mepolizumab (75 mg *i.v.* or 100 mg subcutaneously (*s.c.*), 4 weekly) *versus* placebo; and MUSCA [4], a 24-week study of mepolizumab 100 mg *s.c.*, 4 weekly) *versus* placebo. All studies recruited patients with two or more exacerbations in the previous year and evidence of eosinophilic airway inflammation. As similar efficacy on exacerbation rate reduction has been shown across all doses of mepolizumab, these were combined.

Patients completed an electronic daily diary card during treatment, including a six-point symptom score assessing asthma symptoms in the previous 24 h (0: no symptoms; 5: worst symptoms) and a best-of-three morning peak expiratory flow (PEF) (in litres per minute). Exacerbations were defined as worsening symptoms requiring rescue oral corticosteroids (OCS) for ≥3 days. Exacerbations with ≥20 days of diary data in the period from 14 days prior to and after starting OCS (defined as day 0) were included in the analysis. Change from day −14 (*i.e.* 14 days prior to starting OCS) in PEF and symptom score were compared between treatment arms using unpaired t-tests. Rate of change in endpoints in the 7 days after OCS initiation (*i.e.* days 0–7) were compared between treatments using linear regression. For individuals with multiple events, within-subject means were derived prior to analysis.

During the studies, 322 (52%) patients on placebo and 419 (37%) patients on mepolizumab experienced one exacerbation or more. Sufficient diary card PEF data were available for 1021 exacerbations (473 occurring in 247 subjects on placebo and 548 in 331 subjects on mepolizumab). Sufficient symptom data were available for 1026 exacerbations (476 occurring in 248 patients on placebo and 550 in 332 patients on mepolizumab). Baseline mean±sd characteristics were similar between mepolizumab and placebo respectively: age 50±12 *versus* 50±13 years, female sex 63% *versus* 66%, body mass index 28.9±6.1 *versus* 28.4±6.5 kg·m^−2^, pre-bronchodilator forced expiratory volume in 1 s (FEV_1_) 57.3±17.2% *versus* 59.3±16.6% of predicted, geometric mean blood eosinophil count 0.23±1.1 *versus* 0.34±1.0 cells per mm^3^, day −14 PEF 245±108 *versus* 262±107 L·min^−1^ and day −14 symptom score 1.4±1.4 *versus* 1.6±1.3.

Exacerbations on placebo were associated with a larger mean drop in PEF compared to mepolizumab at day 0 *versus* day −14 (−39.0 *versus* −29.5 L·min^−1^, mean difference (95% CI) 9.5 (0.6–19.6) L·min^−1^) (figure 1), representing a 15% and 12% decrease from day −14 respectively. PEF from day 0 to 7 recovered at 4.8 and 3.6 L·min^−1^ per day (mean difference 1.2 (−0.2–2.6) L·min^−1^), representing a percentage of the total fall in PEF recovered per day of 12% for both treatments. The mean increase in daily symptom score at day 0 compared to day −14 was 0.64 and 0.68 points for placebo and mepolizumab (mean difference 0.03 (−0.15–0.22)) (figure 1), representing an increase of 44% and 45% from day −14 respectively. The rate of recovery of symptom scores from day 0 to 7 was 0.49 *versus* 0.40 points per week (mean difference 0.09 (−0.01–0.19) points per week), representing 19% and 13% of the total drop recovered per day for the placebo and mepolizumab groups (figure 1). The profile of symptoms and PEF from day −14 to 14 did not differ by mepolizumab dose.

**FIGURE 1:**
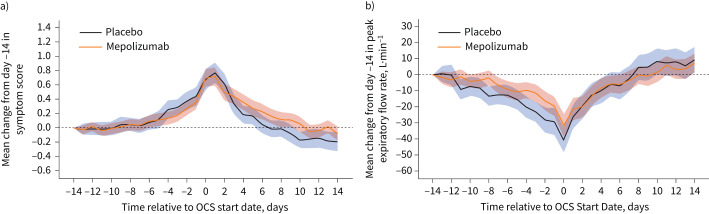
Changes in a) symptom score and b) peak expiratory flow by day before and after oral corticosteroids (OCS) were started (day 0).

In patients treated with mepolizumab, exacerbations resulted in shallower PEF reduction but similar increases in symptom scores. In contrast, the rate of recovery following treatment with prednisolone was similar for PEF but tended to be slower for symptoms scores. These findings suggest that some exacerbation events occurring in patients treated with mepolizumab are different and less prednisolone responsive. This supports the exacerbation heterogeneity in the MEX study. MEX characterised the exacerbations on mepolizumab and identified two groups: an exhaled nitric oxide fraction (*F*_ENO_) and sputum eosinophil high group, and a *F*_ENO_ and sputum eosinophil low group who were often pathogen positive [2]. However, MEX did not assess clinical recovery after treatment with prednisolone.

In patients with COPD, noneosinophilic exacerbations are associated with a shallower fall in lung function and a slower symptom recovery after treatment with prednisolone compared to eosinophilic events [5]. Similarly, our findings suggest a distinct phenotype of exacerbation not prevented by mepolizumab treatment and less responsive to prednisolone. The presence of a mepolizumab-unresponsive exacerbation type is supported by results from two large placebo-controlled studies of mepolizumab in eosinophilic COPD. Exacerbations treated with antibiotics alone were not prevented by mepolizumab treatment, whereas OCS-treated events were prevented in a blood eosinophil-dependent manner [6].

We acknowledge that the changes in PEF at exacerbation and the differences in rate of recovery in symptoms were small. These were *post hoc* exploratory analyses, so we did not perform any hypothesis testing. Our findings should therefore be regarded as hypothesis generating rather than definitive. They provide some support for the view that on-biologic exacerbation mechanisms are heterogeneous. One possibility is that anti-IL-5 treatment shifts the balance to an increased proportion of infective exacerbations, although patients displaying broader epithelial activation may still be prednisolone-responsive [7]. Better characterisation of exacerbations based on their biology might prevent prednisolone use with an unfavourable risk/benefit balance.

There are additional limitations to our study. First, ∼20% of exacerbations were excluded due to insufficient diary card data. This was unlikely to cause bias because these patients had similar baseline characteristics to included patients. Second, we included all doses of mepolizumab as they have similar efficacy. We observed no relationship between mepolizumab dose and the profile of exacerbations, so it is unlikely that higher doses, which reduce sputum eosinophils more completely [1], affect the response to prednisolone. Third, we used mean data from each patient to reduce the possibility of frequently exacerbating patients skewing the analysis, which may be overly conservative. Fourth, our earlier study [8] identified a shallower increase in symptom scores but a similar fall in FEV_1_ between on-mepolizumab *versus* placebo exacerbations, whereas this study showed a reduction in PEF fall but no difference in symptom scores. The use of different measures of symptoms and lung function complicates this comparison. Collectively, both studies imply less severe and prednisolone-responsive events in patients treated with mepolizumab. Fifth, since *F*_ENO_ and blood eosinophils were not measured at exacerbation, we cannot comment on the biological characterisation of the exacerbations. Finally, to account for potential bias and overtreatment with prednisolone, all exacerbations in the DREAM and MENSA clinical trials were corroborated by diary card review. Since a very low percentage of events were rejected, this was not applied to the more recent MUSCA study. Nevertheless, this validation step could have reduced the true between-treatment differences in exacerbation severity.

Prospective studies are needed to determine whether mepolizumab exacerbation events have different inflammatory mechanisms, triggers and oral corticosteroid response [9].

## Data Availability

Anonymised data from the studies listed within this publication and their associated documents can be requested for further research from www.gsk-studyregister.com.

## References

[1] Pavord ID, Korn S, Howarth P, et al. Mepolizumab for severe eosinophilic asthma (DREAM): a multicentre, double-blind, placebo-controlled trial. Lancet 2012; 380: 651–659. doi:10.1016/S0140-6736(12)60988-X 22901886

[2] McDowell PJ, Diver S, Yang F, et al. The inflammatory profile of exacerbations in patients with severe refractory eosinophilic asthma receiving mepolizumab (the MEX study): a prospective observational study. Lancet Respir Med 2021; 9: 1174–1184. doi:10.1016/S2213-2600(21)00004-7 33971168

[3] Ortega HG, Liu MC, Pavord ID, et al. Mepolizumab treatment in patients with severe eosinophilic asthma. N Engl J Med 2014; 371: 1198–1207. doi:10.1056/NEJMoa1403290 25199059

[4] Chupp GL, Bradford ES, Albers FC, et al. Efficacy of mepolizumab add-on therapy on health-related quality of life and markers of asthma control in severe eosinophilic asthma (MUSCA): a randomised, double-blind, placebo-controlled, parallel-group, multicentre, phase 3b trial. Lancet Respir Med 2017; 5: 390–400. doi:10.1016/S2213-2600(17)30125-X 28395936

[5] Bafadhel M, McKenna S, Terry S, et al. Blood eosinophils to direct corticosteroid treatment of exacerbations of chronic obstructive pulmonary disease: a randomized placebo-controlled trial. Am J Respir Crit Care Med 2012; 186: 48–55. doi:10.1164/rccm.201108-1553OC 22447964 PMC3400995

[6] Pavord ID, Chanez P, Criner GJ, et al. Mepolizumab for eosinophilic chronic obstructive pulmonary disease. N Engl J Med 2017; 377: 1613–1629. doi:10.1056/NEJMoa1708208 28893134

[7] Howell I, Yang F, Brown V, et al. Anti-inflammatory effects of oral prednisolone at stable state in people treated with mepolizumab: a proteomic and bulk transcriptomics analysis. medRxiv 2024; pre print [10.1101/2024.02.14.24302812].

[8] Shrimanker R, Pavord ID, Yancey S, et al. Exacerbations of severe asthma in patients treated with mepolizumab. Eur Respir J 2018; 52: 1801127. doi:10.1183/13993003.01127-2018 30464012

[9] Howell I, Mahdi M, Bafadhel M, et al. Recovery of breakthrough asthma attacks treated with oral steroids while on monoclonal antibody therapy: protocol for a prospective observational study (BOOST). JMIR Res Protoc 2023; 12: e46741. doi:10.2196/46741 37351918 PMC10337461

